# Natural infection in anopheline species and its implications for autochthonous malaria in the Atlantic forest in Brazil

**DOI:** 10.1186/1756-3305-6-58

**Published:** 2013-03-07

**Authors:** Ana Maria RC Duarte, Diego M Pereira, Marcia B de Paula, Aristides Fernandes, Paulo R Urbinatti, Andressa F Ribeiro, Maria Helena SH Mello, Marco O Matos, Luís F Mucci, Lícia N Fernandes, Delsio Natal, Rosely S Malafronte

**Affiliations:** 1 Biochemistry and Molecular Biology Laboratory, Superintendency for the Control of Endemic Diseases (SUCEN), Rua Paula Souza 166, São Paulo 01027-000, Brazil; 2 Epidemiology Department, Faculty of Public Health, University of São Paulo, Av. Dr. Arnaldo715, São Paulo 01246-904, Brazil; 3 Zoonosis Control Center, Laboratory for the Identification of and Research into Synanthropic Fauna/Lab-Fauna, Rua Santa Eulália 86, São Paulo 02031-020, Brazil; 4 Culicid Laboratory/SR-03, SUCEN, Pça. Coronel Vitoriano 23, Taubaté 12020-020, Brazil; 5 Protozoology Laboratory, Institute of Tropical Medicine, University of São Paulo, Av. Dr. Enéas de Carvalho Aguiar 470, São Paulo 05403-000, Brazil; 6 Department of Infectious and Parasitic Diseases, Faculty of Medicine, University of São Paulo, Av Dr Arnaldo 455, São Paulo, 01246-903, Brazil

**Keywords:** Autochthonous malaria, Plasmodium, Anophelines, Natural infection, Atlantic forest

## Abstract

**Background:**

A descriptive study was carried out in an area of the Atlantic Forest with autochthonous malaria in the Parelheiros subdistrict on the periphery of the municipality of São Paulo to identify anopheline fauna and anophelines naturally infected with *Plasmodium* as well as to discuss their role in this peculiar epidemiological context.

**Methods:**

Entomological captures were made from May 2009 to April 2011 using Shannon traps and automatic CDC traps in four areas chosen for their different patterns of human presence and incidences of malaria (anthropic zone 1, anthropic zone 2, transition zone and sylvatic zone). Natural *Plasmodium* infection was detected by nested PCR based on amplification of the 18S rRNA gene.

**Results:**

In total, 6,073 anophelines were collected from May 2009 to April 2011, and six species were identified in the four zones. *Anopheles cruzii* was the predominant species in the three environments but was more abundant in the sylvatic zone.

*Anopheles (Kerteszia) cruzii* specimens from the anthropic and sylvatic zones were positive for *P. vivax* and *P. malariae*. *An. (Ker.) bellator*, *An. (Nys.) triannulatus*, *An. (Nys.) strodei*, *An. (Nys.) lu*tzi and *An. (Ano) maculipes* were found in small numbers. Of these, *An. (Nys.) triannulatus* and *An. (Nys.) lutzi,* which were collected in the anthropic zone, were naturally infected with *P. vivax* while *An. (Nys.) triannulatus* from the anthropic zones and *An. (Nys.) strodei* from the transition zone were positive for *P. malariae*.

**Conclusion:**

These results confirm that *Anopheles (Kerteszia) cruzii* plays an important role as a major *Plasmodium* vector. However, the finding of other naturally infected species may indicate that secondary vectors are also involved in the transmission of malaria in the study areas. These findings can be expected to help in the implementation of new measures to control autochthonous malaria in areas of the Atlantic Forest.

## Background

The peculiar epidemiological situation regarding malaria in the Atlantic Forest in the southern and southeastern regions of Brazil is characterized by atypical cases involving asymptomatic or oligosymptomatic individuals, most of whom are infected with *Plasmodium vivax* and live in forested areas; one main mosquito vector, *Anopheles (Kerteszia) cruzii*, whose larval phase develops in bromeliad axils; and the presence of wild monkeys that could act as reservoirs [1-8].

The finding outside the Amazon region of individuals with antibodies against sporozoite and asexual forms of *P. vivax* and *P. malariae* and of a few human cases with low malaria parasitemias reinforces the hypothesis, supported by several authors [1-3], that asymptomatic individuals may act as a source of transmission in the extra-Amazon region. However, the etiological origin of these cases remains unclear and the presence of monkeys naturally infected with *P. brasilianum* and *P. simium* in these areas suggests that wild reservoirs of *Plasmodium* may be involved in the malaria cycle [4-6].

Even though the ecological aspects of *An. (Ker.) cruzii* are well known [9], little information about natural malaria infection in this vector is available. During the ’40s there were reports, based on microscopic observations, of natural infection in specimens of the *Kerteszia* subgenus collected in southeastern Brazil [10]. More recently, *An (Ker.) cruzii* specimens tested by ELISA were found to be infected with the classic and VK247 variants of *P. vivax*[11].

The objective of this research was to describe the epidemiological context in the region where these anophelines are found, an area of Atlantic Forest with autochthonous malaria transmission located in the Parelheiros subdistrict on the periphery of the municipality of São Paulo. In addition, as the region has the largest number of notified cases of the disease in the Atlantic Forest in the state of São Paulo in recent years, the study also sought to identify entomological and epidemiological aspects that may help gain a better understanding of malaria and improve control of the disease in this region.

## Methods

### Study area

**Figure 1:**
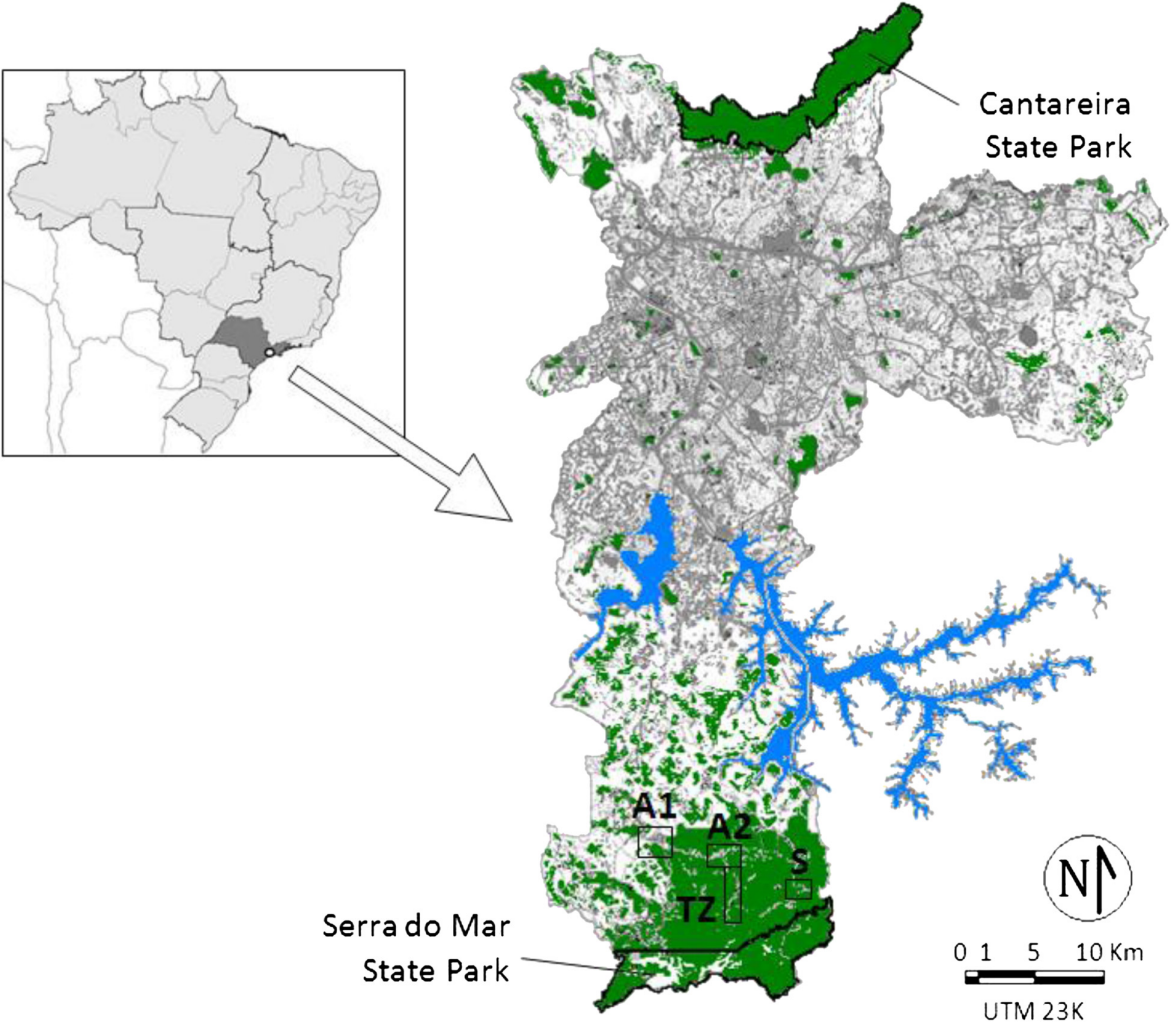
**Study area showing the location of the four zones in the Parelheiros subdistrict: A1 (Anthropic 1 Zone), A2 (Anthropic 2 Zone), TZ (Transition Zone) and S (Sylvatic Zone).** Major state parks (black line), water storage dams (blue), built-up areas (gray), agricultural and unforested areas (white) and forest environment (green) in the municipality of São Paulo are also shown.

The study was performed in three areas in the Parelheiros subdistrict of São Paulo (Engenheiro Marsilac, Embura and Evangelista de Souza), where there were 64 autochthonous cases of malaria in the last five years. To determine the capture points, the following four subdivisions were defined according to the pattern of human occupation, environmental modifications and notifications of autochthonous malaria (Figure 1):

1) Anthropic zone 1 (Universal Transverse Mercator - UTM 23 K 322648/7357521), corresponding to Embura, an urban area with 5,357 inhabitants. This zone, which is the closest to the city, consists of deforested areas with farms, and the main activity is agriculture. In 2007 there were 16 malaria cases, of which only one was symptomatic, and all were treated with the exception of one individual who refused treatment (data from the Embura Health Center).

2) Anthropic zone 2 (UTM 23 K 326439/7355004), corresponding to Engenheiro Marsilac, a small urban center located along the railway line and approximately 5 km from Embura with a population of about 1,015. From 2006 to 2010, 32 cases were notified but only six of the 32 patients presented with symptoms. All 32 individuals were treated (data from the Marsilac Health Center).

3) Transition zone (UTM 23 K 326931/7353093), corresponding to a rural zone with 461 inhabitants that extends from the periphery of Engenheiro Marsilac, where there are scattered houses, to conserved areas of forest where there is little human influence. Fourteen cases were notified from 2007 to 2010, two of whom presented with symptoms. All individuals were treated (data from the Marsilac Health Center).

4) Sylvatic zone (UTM 23 K 333485/7351745), corresponding to Evangelista de Souza, a continuation of the forested area, where there is little or no human influence. The area is about 6 km from the transition zone. There are few dwellings and some trails to waterfalls as well as other areas for sporadic ecotourism activities, such as swimming, fishing and hiking. No cases were notified in this zone in the study period.

### Entomological collections

Fourteen monthly captures, one night per month, were made from May 2009 to June 2010 in the anthropic 1 and sylvatic zones (where the collection points were considered fixed), and seven twice-monthly captures in the anthropic 1, anthropic 2 and transition zones (where the collection points were considered mobile) from January 2011 to April 2011. Two methods were used: CDC light traps with CO_2_ (dry ice) and Shannon traps. The former were installed in open areas, in houses (at floor level) and at the edge of and inside the forest (at ground level and in the canopy at a height of approximately 10 m). Collections with these traps lasted twelve hours, beginning in the evening at twilight. Captures with the Shannon traps, which were used with a gas lamp to attract the mosquitoes, were carried out close to the houses and at the edge of the forest for four hours beginning at dusk.

Specimens were kept in silica gel and identified using the key [12] adopted by the Entomology Laboratory at the Public Health Faculty, University of São Paulo. After identification, they were conserved in isopropanol until they were assayed by PCR to detect natural infection. The abbreviations used here for the genera and subgenera of Culicidae follow the revised nomenclature [13].

### Polymerase chain reaction (PCR)

After collection, female anophelines were assayed by PCR to detect *Plasmodium* infection. DNA was extracted in pools (a maximum of ten specimens/pool) using the Qiagen DNeasy Blood and Tissue kit according to the manufacturer’s protocol. The pools were separated by species, day of capture and type of trap. Amplification of the *Plasmodium* 18S rRNA gene was performed as described by Win et al. [14], who in turn based their protocol on that described by Kimura et al. [15]. The products were electrophoresed in 2% agarose gel and visualized under ultraviolet light.

## Results

In total, 6,703 female anophelines were collected. *An. (Ker.) cruzii* predominated and was found in the four zones. Other species were found in smaller numbers (Table 1) and included specimens from genera *Nyssorhynchus* and *Anopheles*. Forested areas, i.e., the transition and sylvatic zones, had more *An. (Ker.) cruzii* specimens than those occupied by humans (anthropic zones 1 and 2). Despite their low numbers, *Anopheles (Nyssorhynchus) strodei* specimens were found in the four zones.

**Table 1:** **Anopheline females collected in Shannon and CDC traps in four strategic zones in the Parelheiros subdistrict; number of cases of autochthonous malaria between May 2009 and April 2011; and number of pools of *Anopheles *that were positive for *Plasmodium *infection by PCR**

**Study area/(Number of malaria cases-year)**	**Number of specimens collected**	PCR results*/month of capture	
		***P. vivax***	***P. malariae***
**Anthropic 1 Zone (Embura)/(17 – 2007)**			
*An. (Ker.) cruzii*	438	**2** (Dec 2009; Apr 2011)	(−)
*An.(Nys.) triannulatus*	29	**1** (Feb 2011)	**1** (Feb 2011)
*An. (Nys.) strodei*	34	(−)	(−)
**Anthropic 2 Zone (Marsilac)/(33 – 2006–2009)**			
*An.(Nys.) lutzi*	1	**1** (Jan 11)	(−)
*An. (Ker.) cruzii*	57	(−)	(−)
*An. (Nys.) strodei*	1	(−)	(−)
**Transition Zone/(14 – 2007–2010)**			
*An. (Ker.) cruzii*	1,239	**1** (Feb 2011)	(−)
*An. (Nys.) strodei*	47	(−)	**1** (Feb 2011)
**Sylvatic Zone/(0)**			
*An. (Ker.) cruzii*	4,832	**2** (Jul 2009; Aug 2009)	**1** (Dec 2009)
*An. (Nys.) strodei*	20	(−)	(−)
*An. (Ker.) bellator*	4	(−)	(−)
*An. (Ano.) maculipes/pseudomaculipes*	1	(−)	(−)

* Number of pools of *Plasmodium*-positive *Anopheles*. Each pool contained from 2 to 10 specimens.

(−) = negative.

PCR tests to detect *Plasmodium* were carried out for all females. All DNA samples were negative for *P. falciparum*, seven were positive for *P. vivax* and three for *P. malariae.*

Three specimens of *An. (Ker.) cruzii* collected in the sylvatic zone were positive for plasmodia―two for *P. vivax* and one for *P. malariae*―the latter having been collected in a CDC trap in the canopy. In the transition zone, one specimen of *An. (Nys.) strodei* was positive for *P. malariae*, and one specimen of *An. (Ker.) cruzii* was positive for *P. vivax*. It should be noted that forested areas predominate at these collection points (Table 1).

*P. vivax* was detected in *An. (Ker.) cruzii, An. (Nys.) lutzi* and *An. (Nys.) triannulatus* in anthropic zones 1 and 2, which had fewer anophelines and more malaria cases, while *P. malariae* infection was observed in one specimen of *An. (Nys.) triannulatus* (Table 1).

**Figure 2:**
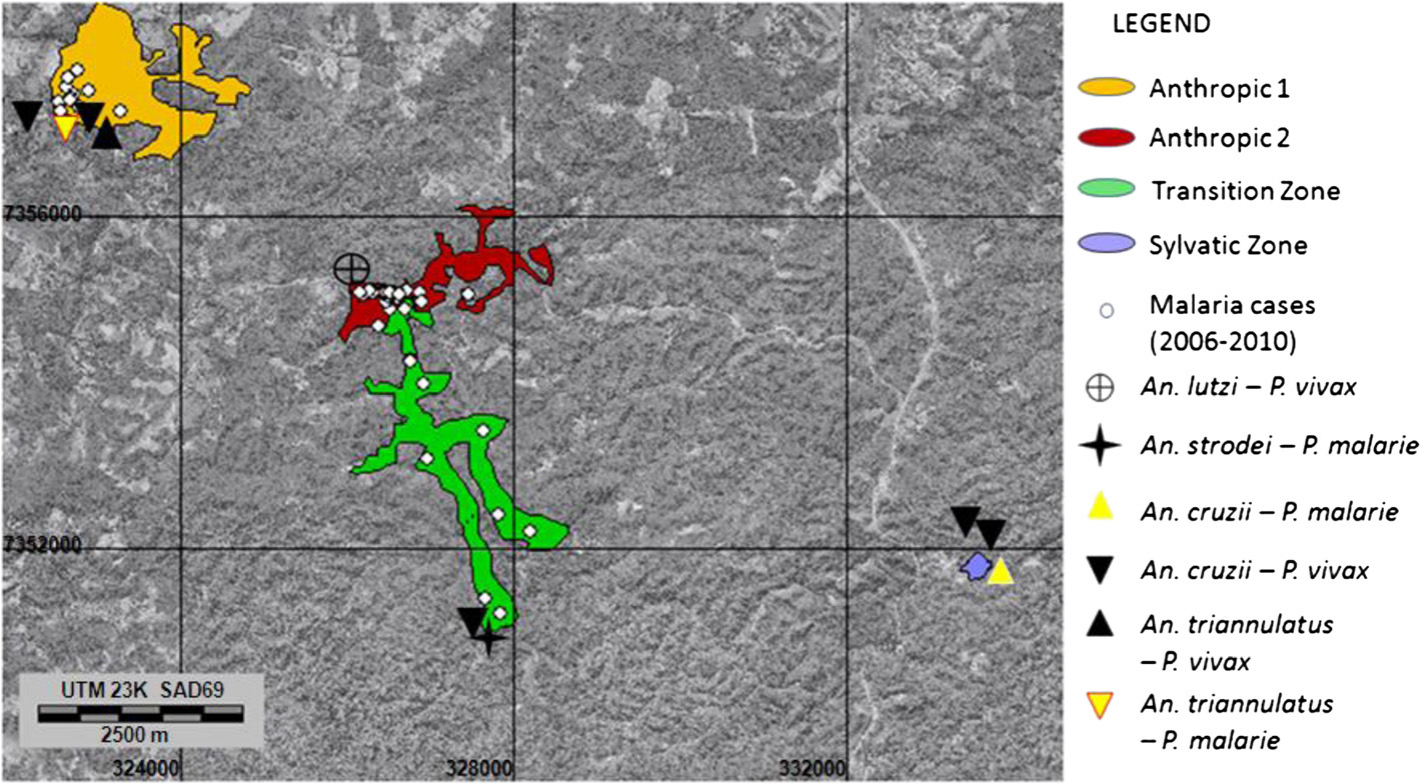
**Distribution of autochthonous malaria cases (between 2006 and 2010) and the sites where *Plasmodium*-positive *Anopheles *were collected in the four zones in Parelheiros subdistrict.** Bright areas indicate urban areas, roads, railroads, agricultural land and pasture; dark gray areas correspond to forest environment. Background: Orthophoto mosaic, scale: 1:5,000; aerial survey of São Paulo Metropolitan Region/EMPLASA (2007).

The capture points, malaria cases and areas where *Plasmodium*-positive anophelines were found are shown in Figure 2.

## Discussion

In the’90s, the bromeliad-malaria epidemiology associated with the Atlantic Forest was discussed by several authors. Four malaria transmission areas in the state of São Paulo are notable in this context: the northern and southern coasts, the *Vale do Ribeira* and areas of metropolitan São Paulo surrounded by the *Serra do Mar*[1,2,8]. Serious social and economic problems have arisen in these areas as a result of human occupation and deforestation, which in some areas may be a critical factor in the increase in the number of cases.

Despite control measures implemented in the past to reduce the number of malaria cases, the Parelheiros subdistrict in southern São Paulo has become an important autochthonous focus of malaria and is notable for the number of cases of the disease there in the last five years. Part of the subdistrict is located in Capivari-Monos Environmental Preservation Area in the Atlantic Forest (Capivari-Monos APA) in the *Serra do Mar*, which is considered a target for ecotourism because of the richness of its fauna, flora and history and its proximity to the metropolitan area.

*An. (Ker.) cruzii* was the predominant species in the study area, and *An. (Ker.) bellator, An.(Nys.) lutzi, An. (Nys.) strodei, An.(Nys.) triannulatus* and *An. (Ano.) maculipes/pseudomaculipes* were found in much smaller numbers*.*

*An. (Ker.) cruzii* and *An. (Ker.) bellator* have been incriminated as vectors of human malaria in the Atlantic Forest [9,10,16], and *An. (Ker.) cruzii* is also a vector of simian malaria in this habitat [4,17].

Subgenus *Kerteszia* is concentrated in exuberant humid forests, where there is a high density of bromeliads [18]. The density of the species in this subgenus varies according to the extent of human disturbance and deforestation [19]. Reflecting this, the greatest number of *An. (Ker.) cruzii* was collected in the sylvatic zone. Some of these were naturally infected by *P. vivax* and *P. malariae*.

In the transition zone, where *P. vivax*–infected anophelines were detected, the population lives on ranches or small farms and is therefore in closer contact with mosquitoes. Between 2007 and 2010, Marsilac Health Center notified 14 cases of vivax malaria.

Fewer *An. (Ker.) cruzii* were collected in the anthropic zones (Embura and Engenheiro Marsilac), where 50 cases were notified from 2006 to 2009 according to the Marsilac Health Center.

As might be expected, these results confirm the importance of *An. (Ker.) cruzii* as the main vector of malaria in the study region; however, other species were also found to be infected by *Plasmodium*. *An. (Nys.) strodei* was positive for *P. malariae* in the transition zone while in the anthropic zones *An. (Nys.) triannulatus* was positive for *P. vivax* and *P. malaria* and only one specimen of *An. (Nys.) lutzi* was positive for *P. vivax*. These findings suggest that other species may play a secondary role in malaria transmission in the Atlantic Forest. Similar results were described in areas surrounded by the Atlantic Forest in the state of Espírito Santo [20].

Although only a few specimens from subgenus *Nyssorrhynchus* were found*,* infection by *Plasmodium* could still be detected, indicating that these anophelines may be important markers of environmental change in areas surrounded by the Atlantic Forest as they are associated with water collections rather than bromeliads. In addition, we found specimens of *An. (Nys.) strodei* and *An. (Nys.) triannulatus* engorged with human blood in the anthropic and transition zones (data not shown).

Although there are still rural communities, the study area is under continuous pressure from urban expansion. The constant exposure of the inhabitants to anopheline bites allied to the distribution of housing over a large geographical area leads to the dispersion of asymptomatic human reservoirs [1,2].

However, the hypothesis that malaria may be a zoonotic disease in the Atlantic Forest has been raised in several studies [4-6,21], and the evidence from the present study underlines the importance of further investigation to determine whether monkeys are acting as malaria reservoirs.

Our findings revealed the presence of *P. malariae*-positive *An. (Ker.) cruzii* in the sylvatic zone and *P. malariae*-positive *An. (Nys.) strodei* and *An. (Nys.) triannulatus* in the anthropic zones, suggesting that *P. malariae* circulates in a simian cycle while concomitantly being transmitted by humans.

Although all the individuals in whom malaria was detected were treated and no new cases have been reported, our results indicate that the sources of infection in the study zones have not been eliminated. For example, the last registered cases of malaria in the anthropic 1 zone were in 2007 but *P. vivax*-positive *An. (Ker.) cruzii* was detected there in 2011; similarly, the most recently registered case in the transition zone was in 2010, but *P. vivax*-positive *An. (Ker.) cruzii* and *P. malariae*-positive *An. (Nys) strodei* were found there in 2011. These findings indicate that silent transmission is being maintained in asymptomatic cases.

Fewer anophelines were collected in the anthropic zones than in the transition or sylvatic zones. However, infections were detected in anophelines from all four zones. The results for disturbed areas suggest that the risk of contracting malaria in these areas may be greater than in wild areas. As our findings are preliminary and the study is descriptive, further investigation is required to correlate the rate of anopheline infection with the incidence of malaria in the population in the study area.

## Conclusion

Our findings point to situations that are novel to areas of the Atlantic Forest where malaria is endemic and indicate that further research is needed to contribute with discussion of new parameters for malaria surveillance and control in this region.

## Competing interests

The authors declare that they have no competing interests.

## Authors’ contributions

All authors were responsible for field collections and the preparation of the manuscript. AMRCD and RSM were responsible for the study design. MBP, AF and AFR were responsible for the identification of mosquitoes. AMRCD and DMP were responsible for the processing of mosquitoes and for analysis. LFM was responsible for the map and for geoprocessing the data. All authors read and approved the final version of the manuscript.
